# 

**Published:** 1913-11-29

**Authors:** 


### CONTENTS.

| | PAGE |
|---|---|
| **Resident Medical Officers and Matrons** ... | 211 |
| **The Lay-Medical Dinner** ... ... ... | 212 |
| **Hospital and Institutional News,** including— Westminster Hospital Site—A Corrugated Iron Hospital—An Appointment "in Camera"?—The New Buildings at the Radcliffe Infirmary, Oxford—Private Sanatoria and Insurance Patients—Some Amusement and a Little Money. | 213 |
| **The Modern Orthopædic Unit** ... ... ... | 219 |
| **The Burden of Routine in Mental Hospitals** ... | 220 |
| **Garden Cities for Consumptives: Administrative Problems** ... ... ... ... ... | 221 |
| **Reports on Halstead and Cromer Cottage Hospitals** ... ... ... ... ... ... By Sir HENRY BURDETT, K.C.B., K.C.V.O. | 225 |
| **The Statistical Report of King Edward's Hospital Fund** ... .. ... ... ... | 227 |
| **The Worcester General Infirmary** ... ... | 228 |
| **Proposed New Regulations as to Medical Benefit** ... ... ... ... ... ... | 229 |
| **Mission Hospitals of the Empire: Their Achievement and Position To-day** ... | 231 |
| **The Editor's Letter-Box** ... ... ... ... Refusal of Benefits—A Patient's Appreciation. | 233 |
| **West Ham and Eastern General Hospital Dinner** ... ... ... ... ... ... | 234 |
| **Sir Felix Schuster on Hospital Finance** ... | 234 |
| **The Latest Hospital History** ... ... ... | 234 |
| **Personalia** ... ... ... ... ... ... | 235 |
| **Gifts and Bequests** ... ... ... ... ... | 235 |
| **Obituary** ... ... ... ... ... ... ... | 235 |
| **The Non-Panel Movement** ... ... ... ... | 236 |
| **The Institutional Worker** ... ... (Suppl.) The Ordeal of a Candidate for a Hospital Secretaryship. | 1 |
| **Our Bureau of Information** ... ... ,, | 2 |
| **Buildings Contemplated and in Progress** ,, | 3 |
| **The Book Market** ... ... ... ,, | 3 |

